# Combined nicotine patch with gum versus nicotine patch alone in smoking cessation in Hong Kong primary care clinics: a randomised controlled trial

**DOI:** 10.1186/s12889-019-7634-z

**Published:** 2019-10-16

**Authors:** Maria K. W. Leung, Dan Bai, Benjamin H. K. Yip, M. Y. Fong, Petty M. H. Lai, Phoebe Lai, Irene S. Y. Lai, Zoe H. W. Lam, Andrew T. F. Leung, Dorothy K Y To, M. T. Wong, T. K. Wong, David V. K. Chao

**Affiliations:** 1 Department of Family Medicine, New Territories East Cluster, Hospital Authority, Hong Kong, China; 2 Division of Family Medicine and Primary Health Care, Chinese University of Hong Kong, Hong Kong, China; 3 Department of Family Medicine and Primary Health Care, Kowloon East Cluster, Hospital Authority, Hong Kong, China; 4 Occupational Therapy Department, Tuen Mun Hospital, New Territories West Cluster, Hospital Authority, Hong Kong, China; 5 Department of Family Medicine, Kowloon Central Cluster, Hospital Authority, Hong Kong, China; 6 Department of Family Medicine, Hong Kong East Cluster, Hospital Authority, Hong Kong, China; 7 Department of Family Medicine, New Territories West Cluster, Hospital Authority, Hong Kong, China

**Keywords:** Effectiveness, Combined nicotine replacement therapy, Nicotine patch, Smoking cessation, Randomised controlled trial

## Abstract

**Background:**

The prevalence of daily cigarette smoking has dropped to 10% in Hong Kong (HK) in 2017, however, smoking still kills 5700 persons per year. Studies suggest that abstinence rates are higher with combined NRT than single NRT, although local data on safety and benefits of combined NRT are lacking. The aim of this study is to compare the effectiveness of combined NRT with single NRT among HK Chinese.

**Methods:**

This is a one-year, two-arm, parallel randomised trial. Five hundred sixty smokers, who smoked ≥10 cigarettes/day for ≥1 year, were randomized to combined and single NRT. Combined NRT group received counseling and nicotine patch & gum. Single NRT group received counselling and nicotine patch. Primary outcome was abstinence rate measured as self-reported 7-day point prevalence with CO validated at 52 weeks. Secondary outcomes included smoking abstinence rates at 4, 12, & 26 weeks. Crude odds ratio and *p*-value were reported from logistic regression without adjustment; for trend analysis, adjusted odds ratio (AOR) and p-value were reported from Generalized Estimating Equation (GEE) (controlling for time). All AORs were adjusted for age, sex, baseline CO and clusters.

**Results:**

Abstinence rates at 4, 12, 26 and 52 weeks were all higher in the combined NRT group (35.8, 21.9, 16.8, 20.1%) compared with the single NRT group (28, 16.8, 11.2, 14.3%). At 4 weeks, combined NRT group was more likely to quit smoking (OR 1.43, 95% CI, 1.00 to 2.05) than the single NRT group. From GEE analysis, combined NRT group had a significantly higher abstinence rate (23.6%) than the single NRT group (17.6%) across repeated measures at all-time points. Combined NRT group was more likely to quit smoking (OR 1.43, 95% CI, 1.15 to 1.77). No significant difference in the side effect profile was detected between groups.

**Conclusions:**

Smokers given 8 weeks of combined NRT were more likely to quit smoking at 4, 12, 26 and 52 weeks compared with single NRT. Combined NRT was as well tolerated as single NRT and it should be further promoted in our community.

**Trial registration:**

NCT03836560 from ClinicalTrial.gov, 9 Feb 2019.

## Background

According to Hong Kong Thematic Household Survey Reports, although the prevalence of current daily smokers among aged 15 and over has dropped from 15.3% in 2006 [1] to 10.8% in 2017 [2], smoking still kills 5700 persons per year and contributes to 14% of all deaths from non-communicable diseases [3–5]. In Hong Kong, Hospital Authority is one of the major service providers for smoking cessation. The target recipients of our smoking cessation service are primarily patients attending public general out-patient clinics (GOPCs) for management of chronic illnesses such as hypertension and diabetes mellitus. Thus, enhancement in smoking cessation in our patients would be crucial in improving their medical conditions [6].

In 2012, the pharmacological treatments provided from our clinics were mainly single nicotine replacement therapy and varenicline. Yet, many side effects have been reported with varenicline, including insomnia, nausea and abnormal dreams [7–12]. One local study showed 20% of patients on varenicline experienced gastrointestinal upset, headache, or dizziness [13]. Due to the fear of side effects, many of our patients preferred nicotine replacement therapy to varenicline. Over the last decade, many studies [14–31] had been carried out to compare the effect of monotherapy with combined nicotine replacement therapy (NRT). Combined NRT is believed to provide a stable baseline nicotine level by means of nicotine patch plus intermittent usage of short acting NRT e.g. gums, lozenges or inhalers for withdrawal symptoms. Cochrane meta-analysis in 2013 [23] and database review 2019 [32] both showed that combined NRT were better than monotherapy. Several studies have shown that combined NRT is associated with lower withdrawal scores [15] and higher 6-month abstinence rates (26.9 to 36.9%) when compared with monotherapies (19 to 23%) [16–19]. Indeed, combined NRT has also been shown to be as well tolerated as monotherapy [20].

In Hong Kong, there have been very few data on combination NRT use. One observational study [33] on efficacy of various modalities of nicotine replacement therapy did not show a superior effect of combined NRT to monotherapy on 7-day point prevalence abstinence rates at 26 and 52 weeks when compared with counselling alone. As there has been no previous randomized controlled trial on comparing the effectiveness of combined NRT with single NRT in our locality, this study serves as the first territory wide study in Hong Kong.

### Objective

The aim of this study is to compare the effectiveness of combined NRT with single NRT on smoking cessation. The results of this study can further enhance the strategy in smoking cessation in Hong Kong,

### Key messages

Smokers given 8 weeks of combined NRT had higher abstinence rates at 4, 12, 26 and 52 weeks compared with single NRT. Combined NRT group was 1.43 times more likely to quit smoking than single NRT group. Combined NRT was as well tolerated as single NRT and should be further promoted in smoking cessation in our community.

## Methods

### Study design

**Fig. 1:**
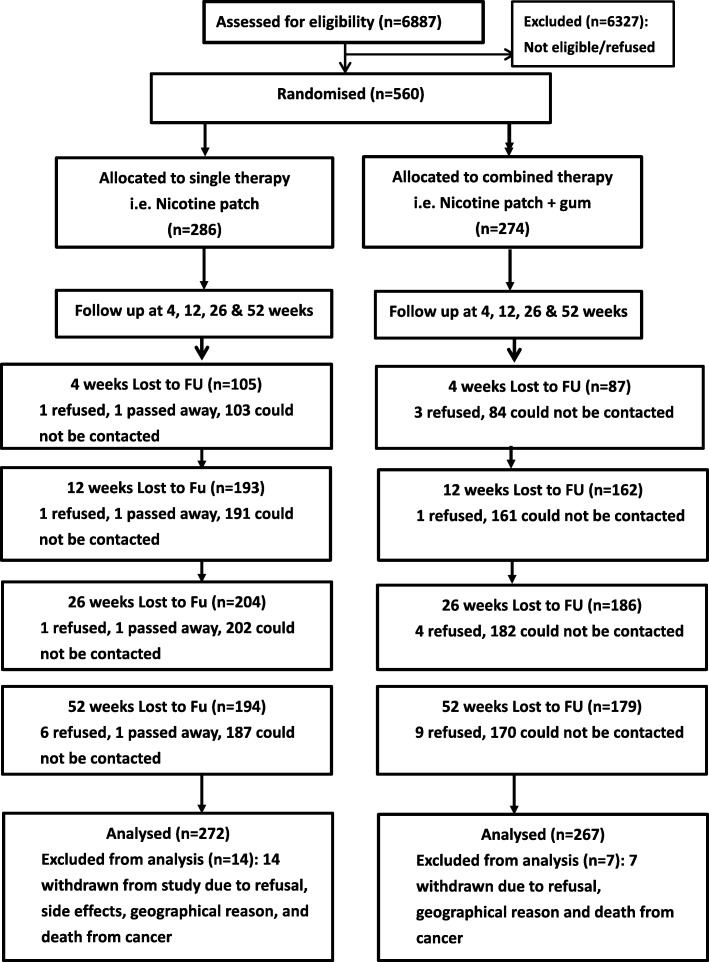
CONSORT workflow diagram

This study utilized an open label, parallel randomized controlled design with two treatment arms among chronic smokers in Hong Kong. The CONSORT flow chart [34] of the study is presented in Fig. 1. Participants of this study were chronic smokers from 20 public primary clinics in Hospital Authority across five clusters: Hong Kong East Cluster (HKEC), Kowloon Central Cluster (KCC), Kowloon East Cluster (KEC), New Territories East Cluster (NTEC) and New Territories West Cluster (NTWC). Written consent was obtained from participants.

### Inclusion criteria

Current smokers, who smoke 10 or more cigarettes a day for at least 1 year, were recruited from 20 primary care clinics across Hospital Authority.

### Exclusion criteria

Smokers with unstable angina, severe cardiac arrhythmia, recent acute myocardial infarction or cerebrovascular accident in preceding 3 months [35], below 18 years old, being pregnant or on breast-feeding, unable to use gum, with a previous history of failure to NRT were excluded from the study.

### Sample size calculation

According to a meta-analysis in 2008 [19], smoking cessation rates (26 weeks post quit) for combined nicotine therapy was about 36.5% and that of nicotine patch was 23.4%. Based on the sample size calculator provided from Centre for Clinical Research and Biostatics [36–38], the Chinese University of Hong Kong, the sample size required with equal allocation (*r* = 1) to achieve a 90% power (*β* = 0.1) at *α* = 0.05 will be n1 = n2 = 252. Considering that there would be a 10% drop-out, the effective sample size was 280 smokers per arm.

### Randomisation [39, 40]

Doctors and nurses from the involved clinics referred motivated patients to smoking cessation counselors at the sites. In the initial assessment, smoking cessation counselor assessed their smoking history. When the patient met the inclusion criteria, smoking cessation counselors would then explain the research to the patient and obtained written consent from the patient for enrollment. A statistician who was not involved in the statistical analysis independently randomized participants by using a predetermined random table generated by Microsoft Excel 2002. The randomization number generated was assigned to one of the two treatments: combined nicotine replacement therapy or single nicotine replacement therapy. Block randomization approach was conducted to control the two arms with an 1:1 ratio in each clinic. The counsellor who had been concealed from the randomisation and allocation sequence, then assigned the patient to their specified intervention according to the allocated number. Once the patient was started the treatment, they would then be followed up at 1 week, 4 weeks, 12 weeks, 26 weeks and 52 weeks after quit day.

### Assessment

Patients were seen at baseline for assessment, and then at 4 weeks, 12 weeks, 26 weeks and 52 weeks. Study medication was given at baseline and at week 4. In baseline assessment, smoking history including daily cigarette consumption and past quitting method, past medical health, drug history and allergy would be obtained. In follow up visits patients were assessed on nicotine withdrawal symptoms, carbon monoxide level, side effects from treatment and medication compliance. Counselling would be given in all follow up visits.

### Pharmacological interventions

Patients were randomised to open-label combined NRT or single NRT with nicotine patch for smoking cessation. Nicotine replacement therapy (NRT) was given for 8 weeks in both arms. Intervention consisted of counseling and combined NRT of nicotine patch and gum. For those smoking 20 or more cigarettes per day before quitting, the NRT patch regimen was 4 weeks of 21 mg patches, then 2 weeks of 14 mg patches, followed by 2 weeks of 7 mg patches. For those smoking 10 to 19 cigarettes per day before quitting, the NRT patch regimen was 4 weeks of 14 mg patches, followed by 4 weeks of 7 mg patches. Two milligram nicotine gum was used once every 1 to 2 h when required. Usual care involved counseling and single NRT of nicotine patch. NRT patch regimen used in usual care was the same as that in intervention group.

### Counselling

Counselling was based on the 2013 service framework for smoking counselling and cessation programme from Hospital Authority [41]. Counsellors were registered nurse who had undergone training in smoking cessation organized by Hospital Authority.

### Outcome measures

Participant’s quit status was confirmed by self-reporting of 7-day point-prevalence abstinence at 52 weeks (defined as not smoking during the 7 days preceding the 52-week follow up) with confirmation by biochemical confirmation via exhaled carbon monoxide (CO) level. Biochemical confirmation of abstinence [42] required a carbon monoxide level of 6 ppm or lower, which is based on the operation manual of the CO meter [43]. Abstinent participants had to report not smoking at all in the last week and have a CO level of ≤6 ppm. All others, including those missing at follow-up, were considered as smoking, a definition as recommended by a research guideline [44]. Primary outcome of this study was smoking abstinence rate at 52 weeks and secondary outcomes included smoking abstinence rates at 4, 12 and 26 weeks. Demographic characteristics, including sex, age, marital status, social-economic status (education, work status), previous smoking status (Fagerstrom score, pack-years, baseline CO level) and smoking related health conditions (respiratory disease, cardiovascular disease) were collected at baseline. Side effects from nicotine replacement therapy such as skin rash, gastrointestinal discomfort, would also be recorded and analysed.

### Data collection

In order to safeguard patient confidentiality, patients’ data was collected by counselors and was stored as computerized records in Clinical Management System (CMS) which could only be accessed by the doctors and smoking cessation counselors who had access rights to CMS in the centre. CO level was measured by counsellors at each visit and data was entered in CMS.

### Data analysis

The statistician was blinded during the analysis process. Data collected was analysed on an intention-to-treat basis. Baseline characteristics were reported and compared by treatment groups, two-sample t test was conducted for continuous variables and Chi-square test for categorical variables. At each visit, crude odds ratio (OR) (combined NRT vs. single NRT) with 95% confidence interval (CI) was reported, simple logistic regression was utilized without adjustment first, and then adjusted for potential confounders, age, sex, baseline CO level and cluster site of the subject recruitment. The overall treatment effect (combined NRT vs. single NRT) over the study period was estimated by Generalized Estimating Equation (GEE) [45]. In the GEE model, time (repeated measures at 4, 12, 26, and 52 weeks) was included as a continuous covariate, adjusted odds ratio (AOR) was reported and treatment-time interaction was tested. Potential confounders were also adjusted for in the GEE model. Statistical significance level was set at two-sided *p* < 0.05 for all tests. Analysis was conducted by R version 3.2.2 [46]. Side effects from NRT were also recorded from both groups. Chi-squared test was used to compare the difference.

## Results

### Patient recruitment and characteristics

A total of 560 smokers were recruited from Feb 2015 to Jan 2017 and they were all followed up for 1 year after quit day. They were randomised to either combined NRT group (274 participants) or single NRT group (286 participants) for smoking cessation from 20 clinics in five clusters (112 each). 21 (3.75%) smokers withdrew from the study, 14 (4.9%) from the single NRT group and 7 (2.6%) from the combined group (Fig. 1. CONSORT workflow diagram). There was no significant difference between the two groups (*p* = 0.138). At baseline, most of the participants were male (477, 85.2%) and married (424, 75.7%), the average age was 50.48 years. Regarding the background smoking history, on average, the participants smoked 18.56 cigarettes per day for 32.05 years, i.e., 29.81 pack years. In terms of nicotine dependence, the average Fagerstrom score was 5.71 (moderate), and average baseline CO level was 20.59, for both groups. 56.6% of smokers were heavy smokers in both the single and combined NRT groups. About one third (185, 33.0%) of the participants had cardiovascular (CV) disease, 20.2% had endocrine disease, and 5.9% had respiratory disease. Overall, no significant differences were detected between single NRT and combined NRT groups for all characteristics in Table 1.

**Table 1:** Baseline characteristics by treatment groups

Characteristic	Single NRT (*N* = 286)	Combined NRT (*N* = 274)	*p*-value
Age (in years), mean (SD)	50.56 (12.08)	50.41 (12.30)	0.884
Male, No. (%)	236 (82.5)	241 (88.0)	0.091
Marital Status, No. (%)			0.386
Single	48 (16.8)	37 (13.5)	
Married	208 (72.7)	216 (78.8)	
Divorced/Separated	23 (8.0)	17 (6.2)	
Widowed	7 (2.4)	4 (1.5)	
Education, No. (%)			0.565
No formal schooling	3 (1.0)	7 (2.6)	
Primary	60 (21.0)	61 (22.3)	
Secondary	203 (71.0)	187 (68.2)	
Tertiary	20 (7.0)	19 (6.9)	
Work status, No. (%)			0.310
Unemployed	61 (21.3)	47 (17.2)	
Full time	181 (63.3)	188 (68.6)	
Part time	16 (5.6)	8 (2.9)	
Retired	20 (7.0)	24 (8.8)	
Housewife	8 (2.8)	7 (2.6)	
Payment status, No. (%)			0.506
Entitled patients	234 (81.8)	220 (80.3)	
Waivers	18 (6.3)	24 (8.8)	
Hospital Authority staff/Civil servants	29 (10.1)	28 (10.2)	
Others	5 (1.7)	2 (0.7)	
Cluster, No. (%)			0.946
HKEC	56 (19.6)	56 (20.4)	
KCC	55 (19.2)	57 (20.8)	
KEC	61 (21.3)	51 (18.6)	
NTEC	57 (19.9)	55 (20.1)	
NTWC	57 (19.9)	55 (20.1)	
Co-existing diseases			
Respiratory disease, No. (%)	18 (6.3)	15 (5.5)	0.816
CVS disease, No. (%)	105 (36.7)	80 (29.2)	0.072
Endocrine disease, No. (%)	59 (20.6)	54 (19.7)	0.868
Smoking history			
No. of cigarettes/day used, mean (SD)	18.44 (6.90)	18.68 (6.88)	0.683
Heavy Smoker (≥20 cigarettes/day), No. (%)	162 (56.6)	155 (56.6)	0.988
Years smoked, mean (SD)	31.93 (11.94)	32.17 (12.43)	0.817
Pack-years, mean (SD)	29.45 (15.37)	30.18 (16.65)	0.591
Fagerstrom score, mean (SD)	5.57 (2.01)	5.85 (1.79)	0.090
Fagerstrom score, No. (%)			0.347
Low dependence (0–2)	22 (7.7)	12 (4.4)	
Low to Moderate (3, 4)	58 (20.3)	51 (18.6)	
Moderate (5–7)	160 (55.9)	161 (58.8)	
High dependence (8+)	46 (16.1)	50 (18.2)	
Baseline CO level (ppm), mean (SD)	20.36 (12.03)	20.83 (12.49)	0.652

Note: *NRT* Nicotine replacement therapy, *SD* Standard deviation

For all continuous variables, mean and SD were reported and *p*-values were calculated from two sample t-test; for all categorical variables, number and proportion were reported and *p*-values were calculated from Chi-square test

### Primary outcome

Smoking abstinence rate was 14.3% for single NRT group and 20.1% for combined NRT group at 52 weeks [odd ratio (OR) =1.50, 95% CI 0.97 to 2.35] (Table 2). Over the study period, participants in combined NRT group were significantly more likely to quit smoking than those in single NRT group (OR = 1.43, 95% CI 1.16 to 1.76). After adjusting for potential confounders, minor decrease of treatment effect at 52 weeks (AOR = 1.49, 95% CI 0.95 to 2.36) and the overall treatment effect (AOR = 1.43, 95% CI 1.15 to 1.77) was detected for primary outcome.

**Table 2:** Test for Treatment Effect on Primary Outcome (i.e. 7-day point prevalence, biochemically verified) on abstinence rate

Outcome	Single NRT (*n* = 286)			Combined NRT (*n* = 274)			OR (95% CI)	*p*-value	AOR (95% CI)	*p*-value
	No. of Success	Abstinence Rate^a^ (%)	Available No.	No. of Success	Abstinence Rate^a^ (%)	Available No.				
4 weeks	80	28.0	286	98	35.8	274	1.43 (1.00, 2.05)	0.048^a^	1.38 (0.95, 2.00)	0.087
12 weeks	48	16.8	286	60	21.9	274	1.39 (0.91,2.13)	0.130	1.37 (0.89, 2.13)	0.153
26 weeks	32	11.2	286	46	16.8	274	1.60 (0.99, 2.62)	0.057	1.56 (0.95, 2.57)	0.080
52 weeks	41	14.3	286	55	20.1	274	1.50 (0.97, 2.35)	0.073	1.49 (0.95, 2.36)	0.086
GEE	201	17.6	1114	259	23.6	1096	1.43 (1.16, 1.76)	< 0.001**	1.43 (1.15, 1.77)	0.001**

^a^Abstinence rate refers to 7-day point prevalence, biochemically verified, abstinence rates

** refers to p value with statistical significance

Notes: *NRT* Nicotine replacement therapy

*OR* Odds ratio. At each visit, crude odds ratio (combined NRT vs. single NRT) and *p*-value was reported from logistic regression without adjustment; for repeated measures, adjusted odds ratio and *p*-value was reported from Generalized Estimating Equation (GEE) adjusted for time

*AOR* Adjusted odds ratio. At each visit, adjusted odds ratio (combined NRT vs. single NRT) and p-value was reported from logistic regression with adjustment for age, sex, baseline CO and cluster; for repeated measures, adjusted odds ratio and p-value was reported from GEE adjusted for time, age, sex, baseline CO and cluster

### Secondary outcomes

**Fig. 2:**
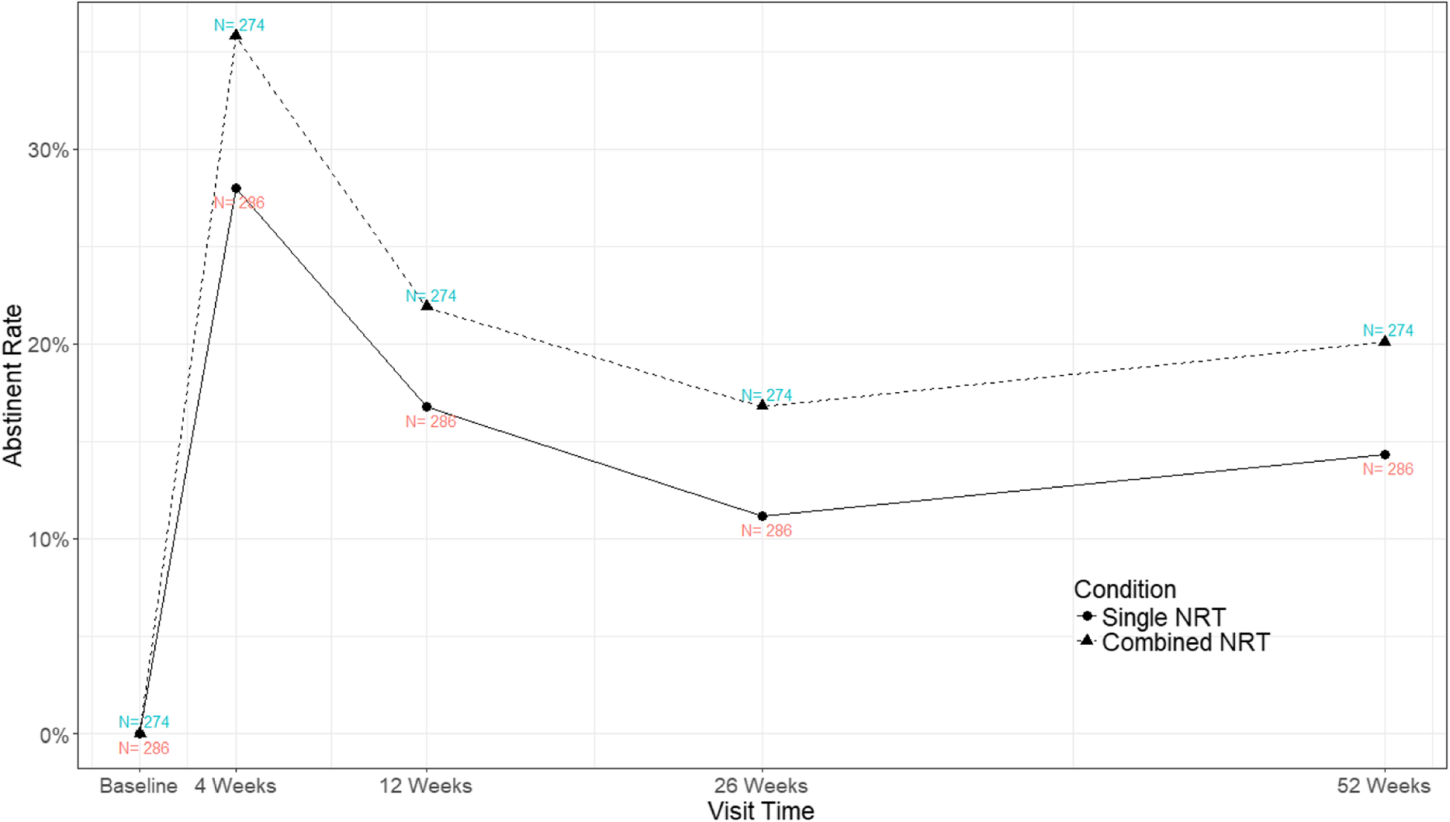
Abstinence rates across visits, by treatment group

For secondary outcomes, the smoking abstinence rate was higher for combined NRT group than single NRT group at each assessment visit i.e. 4, 12, 26 and 52 weeks. Estimates of treatment effect from logistic regression with and without adjustment for age, sex, baseline CO and cluster were reported in Table 2**.** There was significant treatment effect at 4 weeks (OR = 1.43, 95% CI 1.00 to 2.05). As shown in Fig. 2**,** for both groups, treatment effect was optimum at 4 weeks and attenuated with time. There was no crossing between the two lines during the follow-up visits, indicating there was no interaction between treatment and time, which was confirmed by the test on the interaction term in the GEE model (coefficient not reported).

### Subanalysis on the missingness pattern at 52 weeks

**Fig. 3:**
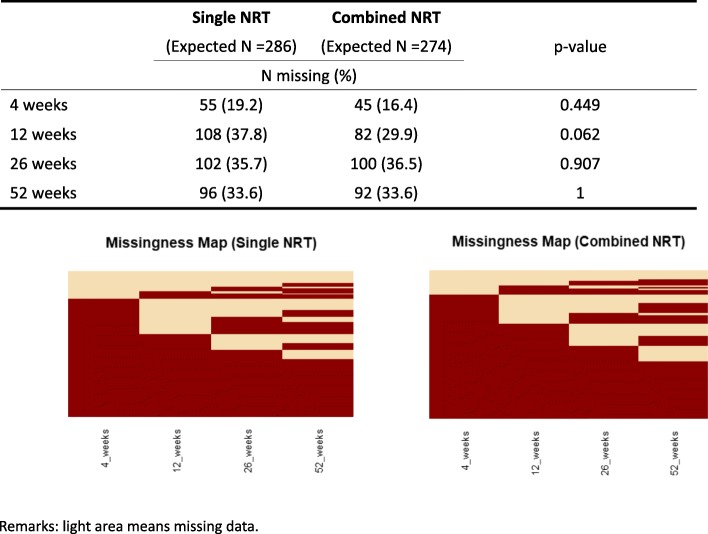
Missing pattern analysis for primary outcome

At 52 weeks, an overall of 66% smokers were lost for follow up. Due to the high loss in data for both groups, a missingness pattern was analysed (Fig. 3), which did not show obvious differences between the two treatment groups across all visits. Sub-analysis showed that baseline age and number of years smoked were significantly associated with missingness at 52 weeks, participants were younger (average age 48.7 vs. 51.4, *p*-value = 0.014) and had less years smoked (30.3 vs. 32.9, p-value = 0.021). Sex, baseline CO level, baseline Fagerstrom score, number of cigarettes smoked per day and compliance (percentage of total patches used) were not significantly associated with missingness at 52 weeks.

### Side effect profile

Overall, 3.4% smokers developed side effects after using NRT. The commonest side effect was skin itchiness and rash (Table 3). In the single NRT group, 12 (4.2%) reported side effects from nicotine patch. In the combined NRT group, 7 (2.6%) reported side effects from NRT. There was no significant difference between the two groups (*p* = 0.315).

**Table 3:** Side effects in both treatment groups

	Single NRT (*N* = 286)	Combined NRT (*N* = 274)	*p*-value
No of patients reported side effects	12 (4.2)	7 (2.6)	0.315
Frequency of side effects			
Abdominal pain	1	0	
Dizziness	1	0	
Dreams	1	0	
Insomnia	1	0	
Palpitation	3	0	
Skin itchiness	3	3	
Skin rash	6	4	
Stomach	1	1	

## Discussion

There have been studies on reviewing the effectiveness of nicotine replacement therapy in Hong Kong. However, there are no randomised controlled trials on comparing single and combined NRT in our locality. This study helps us to understand more on the effect of combined nicotine replacement therapy on smoking cessation in Hong Kong. To our knowledge, this is the first randomised controlled trial on comparing single with combined NRT in our locality. Smokers in both groups had moderate nicotine dependence as shown by their average Fagerstrome score of 5.57 to 5.85 with a baseline CO level of 20 ppm. On average, they smoked 18 cigarettes a day, which was relatively higher than the average daily consumption of cigarette used locally (12.8 cigarettes per day) according to our Hong Kong Thematic Household Survey [4]. In our study, 85% of our smokers were male, which was comparable to the gender pattern as reflected in local survey [4] (85.7%).

Combined NRT showed a higher abstinence rate at all follow up intervals: 4, 12, 26 and 52 weeks (35.8, 21.9, 16.8, 20.1% vs 28, 16.8, 11.2, 14.3%).The abstinence rate at 26 weeks for combined NRT was comparable to the key results from the latest Cochrane database review in 2019 [32] i.e. 15 to 36%. In the Cochrane database review, the short acting NRT used in various studies included spray, gum or lozenge. In comparing with a study on using the same combination i.e. nicotine patch and gum [24], the 26 weeks abstinence rates of combined NRT and single NRT (18.1% vs 12.7%) were very similar to ours (16.8% vs 11.2%). Comparing with those studies which also obtained 52 weeks abstinence rates, the results from our study was also comparable with theirs, 20.1% vs 20.2 to 22.2% [26, 30]. The OR of 52 week abstinent rate of combined NRT of our study was also similar to that reported in the local observational study [33] (OR 1.50, 95% CI 0.97–2.35, vs 1.54, 95%CI 1.22–1.94). Yet, there is a major difference in the quit status confirmation between the two studies. In our study, self-reporting with biochemical verification was required, whereas only self-reporting was needed in the observational study.

The study showed that combined NRT was significantly more superior to single NRT at 4 weeks (35.8% vs 28%). The OR at 4 week was 1.43 (*p* = 0.048, 95% CI, 1.00 to 2.05). From GEE analysis, the overall OR was1.43 (*p* < 0.001, 95% CI, 1.16 to 1.76). The superior effect of combined NRT could be detected as early as 4 weeks after quit date and the effect attenuated with time as confirmed by the GEE model. This result was similar to that noted in the network meta-analysis [23], which showed combined NRT vs NRT patch OR was 1.43 (95% CI, 1.08 to 1.91) at 6 months or longer after quitting.

In general, the use of NRT required a gradual tapering regimen over 8 to 12 weeks. In our study, it was shown that a total of 8 weeks of NRT was efficient enough to show better abstinence rates in sustaining overall smoking cessation. Besides, the low rate of side effects (2.6%) in the combined group also reassured that it is equally well tolerated as single NRT to use for smoking cessation. In some studies, the common side effects such as skin itchiness and rash could occur in up to 10 to 20% [47] of users.

Although the study showed that the abstinence rates for combined NRT group were higher at all follow up intervals than the single NRT group, it did not show statistical significance at 52 weeks. The main reason could be due to the high loss in follow up. In our study, the loss of follow up at 52 weeks was 65 to 68% for combined NRT and single NRT groups, respectively, which had exceeded our planned 10%. Similar loss in follow up was also noted in another study on combined NRT usage with a loss of 59% [30]. There are several reasons why there was such high loss in our study. Firstly, many patients could not be contacted over phone. Secondly, many patients who could be contacted over phone refused to attend clinic again for CO measurement. Thus, only self-reporting result could be obtained. Cost could be an underlying reason for the loss in follow up. Each time they attended the clinic, they had to pay HK$50 for the smoking cessation service. To complete the study, one had to pay HK$250 for the 5 visits within the year. As our outcome measure requires biochemical verification, self-reporting alone would not be sufficient, thus, the quit status could only be considered as smoking. Such high loss in follow up had affected the overall statistical power of the study, by decreasing the statistical power to 54.1 and 47.4% at 26 and 52 weeks, respectively.

In order to minimize the effect, GEE was used for repeated measures and control for time treatment interaction with all available records. This had helped to increase the statistical power increased to over 90%. Further sub-analysis was carried out to review the pattern in missing data and the association with either treatment group. There was no significant difference in the missingness in both treatment groups. In fact, the sub-analysis showed that younger smokers and those with less years of smoking were significantly associated with higher missing data in both treatment groups. Although the underlying reasons why younger smokers tend to be lost in follow up are not known, busy life and work schedule or low perception to health hazards from smoking could be possible. Further studies on young smokers could help to identify better methods for smoking cessation for them.

The study is not without limitations. It was an open-label study, in which both smokers and counsellors were aware of the treatment. This could have, to certain extent, affected the biases of the counsellors and smokers. Also, the study was mainly carried out in public primary care clinics and our smokers were mainly chronic illness patients. We do not know whether chronic illness could have any impact on the efficacy of various smoking cessation methods compared to those without. Further studies on exploring the effect of chronic illness on smoking cessation could be considered.

## Conclusion

Smokers given 8 weeks of combined NRT with nicotine patch and gum had higher abstinence rates at 4, 12, 26 and 52 weeks compared with single NRT and were more likely to quit over the study period. Results also showed that the regimen of combined NRT was as well tolerated as single NRT. Thus, the use of combined NRT should be further promoted as part of the interventions for smoking cessation in primary care clinics in order to help our society to achieve the government’s 2025 target in smoking cessation.

## Data Availability

The datasets generated and analysed during the current study are available upon request.

## Abbreviations

AOR: Adjusted odds ratio
CMS: Clinical Management System
CO: Carbon monoxide
CV: Cardiovascular
GEE: Generalized Estimating Equation
GOPC: General Outpatient Clinic
HKEC: Hong Kong East Cluster
KCC: Kowloon Central Cluster
KEC: Kowloon East Cluster
KWC: Kowloon West Cluster
NRT: Nicotine Replacement therapy
NTEC: New Territories East Cluster
NTWC: New Territories West Cluster
OR: Odds ratio
